# Dietary supplementation of sulfur amino acids improves intestinal immunity to *Eimeria* in broilers treated with anti-interleukin-10 antibody

**DOI:** 10.1016/j.aninu.2022.06.008

**Published:** 2022-06-22

**Authors:** Zhouzheng Ren, Jiakun Yan, Rose Whelan, Xujie Liao, Daniel E. Bütz, Maria K. Arendt, Mark E. Cook, Xiaojun Yang, Thomas D. Crenshaw

**Affiliations:** a College of Animal Science and Technology, Northwest A&F University, Yangling, Shaanxi, China; b Department of Animal Sciences, University of Wisconsin, Madison, WI, USA; c Evonik Operations GmbH, Hanau-Wolfgang, Germany

**Keywords:** Chicken, Coccidiosis, Egg yolk antibody, Interleukin-10, Sulfur amino acid

## Abstract

Oral antibody to interleukin-10 (anti-IL-10) enhances the intestinal immune defense against *Eimeria*. The sulfur amino acids methionine and cysteine (M+C) play essential roles in inducing and maintaining protective immune responses during intestinal infections. Hence, increased dietary M+C may support the anti-IL-10-induced intestinal immunity to *Eimeria*. Broilers (*n* = 640) were arranged in a 2 × 2 × 2 factorial design with 2 levels of each of the 3 main factors: dietary standardized ileal digestible (SID) M+C levels (0.6% or 0.8%), dietary anti-IL-10 supplementation (with or without), and coccidiosis challenge (control or challenge). Briefly, the broilers were supplied with either 0.6% or 0.8% SID M+C, each with or without anti-IL-10 (300 μg/kg), from d 10 to 21. On d 14, broilers from each diet were gavaged with either PBS or *Eimeria*. The resulting *Eimeria* infection induced fecal oocyst shedding and intestinal lesions. Broilers fed 0.8% SID M+C (main effects, *P* ≤ 0.05) had decreased feed-to-gain ratio, increased duodenum and cecum luminal anti*-Eimeria* IgA titers, and decreased fecal oocyst counts, when compared to 0.6% SID M+C. The supplementation of anti-IL-10 (main effects, *P* ≤ 0.05) increased cecum luminal total IgA concentration and decreased cecum lesions. Interactions (*P* ≤ 0.05) were detected for growth performance and cecum luminal IFN-γ. Briefly, the highest body weight gain and feed intake were reached in PBS-gavaged broilers fed 0.8% SID M+C with no anti-IL-10 and in *Eimeria*-challenged broilers fed 0.8% SID M+C with anti-IL-10. In *Eimeria*-infected broilers, anti-IL-10 increased intestinal luminal IFN-γ and body weight gain only at 0.8% SID M+C. Collectively, anti-IL-10 increased intestinal luminal IFN-γ levels, decreased cecum lesions and restored growth only when fed with adequate amounts of sulfur amino acids. Our findings underscore the importance of providing sufficient essential nutrients to support the anti-IL-10 induced immunity against coccidiosis.

## 1 Introduction

*Eimeria*-induced coccidiosis, an enteric disease present in over 90% of floor-reared broilers, causes significant financial losses and antibiotic resistance problems to the poultry industry (Jones et al., 2018). Recent research efforts focused on the pathogenesis of coccidiosis have established a concept that *Eimeria* spp. escapes from host immune attack by stimulating local secretion of interleukin-10 (IL-10) in the intestine of chickens (Arendt et al., 2019a, Arendt et al., 2019b; Cyktor and Turner, 2011). IL-10 is a multi-faceted molecule with a range of reported anti-inflammatory and regulatory functions, which depend on context including but not limited to; timing, tissue, and target cell (Rothwell et al., 2004). If IL-10 is released before a burst of pro-inflammatory activity in response to a pathogen, the ability of the animal to develop adaptive immune responses could be thwarted (Sabat et al., 2010). Persistent infectious viruses, bacteria and helminths have developed mechanisms that up-regulate IL-10 at the beginning of the infectious process to avoid immune detection (Maizels et al., 2004). In some cases, viruses signal for IL-10 to shut down the immune system, and in other cases, infecting organisms send signals to up-regulate IL-10 so the pathogen can avoid immune detection and invade the host (Cyktor and Turner, 2011; Slobedman et al., 2009).

Our laboratory has long been interested in the development of methods to strategically target host molecules in the gastrointestinal tract using oral egg yolk antibodies (Bobeck et al., 2015, 2016; Cook et al., 1999, 2009). We have shown that dietary supplementation of a single-domain egg yolk antibody to IL-10 peptide (anti-IL-10) can alleviate the growth suppression caused by *Eimeria* infection (Arendt et al., 2016; Sand et al., 2016). We also found that an oral antibody to IL-10 receptor 2 provided equivalent benefits to *Eimeria-*infected chickens (Arendt et al., 2019a, Arendt et al., 2019b). Moreover, during the process of *Eimeria* vaccination, a dietary supplementation of anti-IL-10 effectively assisted the chickens to develop early immunity to *Eimeria* spp. (Sand et al., 2016). In studies involving calves, we noticed decreased antibiotic usage when calves were fed diets supplemented with anti-IL-10 in the first d 11 to 14 of life (Raabis et al., 2018). These findings led us to believe that dietary anti-IL-10 supplementation has broad applications.

In addition to immunotherapies like anti-IL-10, the host also requires optimal nutritional support for immunity development. Methionine is the first limiting essential amino acid in most poultry diets worldwide. Therefore, supplementation of methionine to provide a sufficient pool of sulfur amino acids is a widely used practice to ensure adequate growth rates and feed conversion of broilers. Recommendations for standardized ileal digestible (SID) levels of methionine and cysteine (M+C) for optimal growth performance have been well studied and fine-tuned over decades of research (Sauer et al., 2008). However, the effects of graded levels of dietary M+C on immune function in poultry is not well defined. There are some indications that the sulfur amino acids play essential roles in both developing an immune system and mounting an protective immune responses to infection (Grimble and Grimble, 1998; Konashi et al., 2000; Rao et al., 2003; Takahashi et al., 1997). Our previous data repeatedly support that dietary M+C deficiency can delay or impair the development of intestinal immunity to *Eimeria* in broilers (Ren et al., 2020; Tsiagbe et al., 1987a, 1987b). We therefore hypothesized that if the use of anti-IL-10 stimulated earlier immunity to *Eimeria*, then additional benefits may occur by providing optimal sulfur amino acids to support this immune process. To test this hypothesis, an *Eimeria*-challenge study was conducted in broilers fed varied levels of SID M+C in the diet with and without anti-IL-10. Changes in growth performance and intestinal and plasma immune responses were analyzed. Our objective was to determine if supplementation of dietary sulfur amino acids to ensure nutritional requirements are met, would increase immunity to *Eimeria* in broilers treated with anti-IL-10 egg yolk antibody.

## 2 Experimental methods

### 2.1 Animals, diets, and experimental design

Six hundred and forty male broilers (Ross × Ross 308; 1-d-old; with no coccidiosis vaccination; purchased from Welp Hatchery, Bancroft, IA) were randomly allotted to 80 pens (8 broilers per pen) and housed in a battery brooder with raised wire floors. From d 1 to 10 of age, all the broilers were fed with a previously described, standard starter diet (Ren et al., 2020). On d 11, the 80 pens were randomly assigned to 1 of 8 treatments (8 broilers per pen, 10 pens per treatment) in a 2 (dietary SID M+C levels) × 2 (dietary anti-IL-10 supplementation) × 2 (coccidiosis challenge) factorial arrangement. Briefly, the broilers were supplied with either 0.6% or 0.8% SID M+C, each with or without anti-IL-10 (300 μg antibody per kg of diet), from d 10 to 21. On d 14, broilers from each diet were orally gavaged with either phosphate-buffered saline (PBS) or a commercial coccidiosis vaccine (a mixture of low virulent *Eimeria cervuline*, *Eimeria maxima*, and *Eimeria tenella* oocysts; Advent, Lincoln, NE) at 100 × vaccine dose. Changes in dietary SID M+C levels were achieved by the supplementation of DL-methionine (MetAMINO Evonik Operations GmbH, Germany) to a basal diet. Composition of the corn-soybean meal basal starter and grower diet are identical to an earlier publication (Ren et al., 2020) and can additionally be found in Appendix Table 1. The analyzed amino acid levels and anti-IL-10 concentrations of the experimental diets are shown in Appendix Table 2. The coccidiosis challenge model has been shown to induce a subclinical coccidiosis challenge without high mortality risk (Ren et al., 2020; Sand et al., 2016). The broilers had free access to feed and water during the whole experimental period.

Body weight gain, feed intake and feed-to-gain ratio of the broilers were calculated/recorded at the following intervals: from d 11 to 14, from d 15 to 21, and from d 11 to 21 of age. At the end of the study, fresh excreta samples from each pen were collected and the McMaster technique (Arendt et al., 2016) was used for the quantification of *Eimeria* oocysts. At the same time, 1 bird per pen was randomly selected and euthanized for the collection of blood (centrifuged for plasma separation) and intestinal lumen contents (duodenum, jejunum and cecum). Meanwhile, the intestinal coccidiosis lesion scores of sampled broilers were blindly determined by an experienced Doctor of Veterinary Medicine using a well-documented scoring system (Johnson and Reid, 1970), ranging from a score of 0 (no gross lesion) to 4 (severe gross lesions).

### 2.2 Egg yolk antibody preparation

An antigenic chicken IL-10 peptide (val-leu-pro-arg-ala-met-gln-thr, vlpramqt) was synthesized by GeneScript (Piscatawy, NJ), glutaraldehyde-conjugated to bovine gamma globulin (BGG, Sigma, St. Louis, MO), and prepared as a vaccine using previously described procedures (Ren et al., 2017). Laying hens were intramuscularly injected (Ren et al., 2017) with either the IL-10 peptide vaccine or a control vaccine consisted of only glutaraldehyde treated BGG and adjuvants. The vaccination induced an anti-IL-10 antibody which has been shown to effectively neutralize intestine luminal IL-10 in chickens (Arendt et al., 2016; Sand et al., 2016). Eggs containing either control or anti-IL-10 antibodies were collected starting at 21 d after the primary injection and the yolks were separated and dried by lyophilization (to produce egg yolk powder). The egg yolk powder was subjected to a lab-developed enzyme-linked immunosorbent assay (ELISA, see procedures below) for the quantification of anti-IL-10 antibody concentrations. As no anti-IL-10 antibody was detected in egg yolk powder from control vaccinated laying hens, the IL-10 peptide vaccinated laying hens had 732 μg anti-IL-10 antibody in per g of egg yolk powder. Thus, either control or anti-IL-10-containing egg yolk powder was added into the aforementioned experimental diets at a level of 410 mg/kg to create a dietary difference of 0 vs 300 μg/kg anti-IL-10 antibody.

### 2.3 ELISA assays

An ELISA was developed for the quantification of anti-IL-10 antibody concentration in the freeze-dried egg yolk powder and the experimental diets. Anti-IL-10 antibody was extracted from the samples by the addition of acidified PBS (pH = 5; 0.1 g egg yolk powder in 1.9 mL PBS; 1 g feed in 2 mL PBS) for 12 h at 4 °C. Samples were then centrifuged (1,500 × *g*, 10 min, 4 °C). The supernatant was extracted and diluted in 1% milk powder (egg yolk powder supernatant, 1:100; feed supernatant, no dilution) and subjected to the anti-IL-10 quantification ELISA analysis. Affinity purified egg yolk anti-IL-10 was diluted (in 1% milk powder) to 425, 212.5, 106.25, 53.125, 26.5625, 13.28125, 6.640625 and 0 (blank, 1% milk powder only) ng/mL to produce a standard curve. The standards, blanks, and diluted samples were added into the wells at 100 μL/well. The IL-10 peptide (vlpramqt) was conjugated to ovalbumin (OVA) and coated to Costar EIA/RIA 96-well plates (Corning Inc., Corning, NY) at 100 μg peptide/plate. Detailed conditions for plate coating, washing, blocking, incubation and detection, and the source and concentration of secondary detection antibody were the same as previously reported (Ren et al., 2020).

The presence of total IgA (T-IgA), anti-*Eimeria* IgA, IL-10 and interferon gamma (IFN-γ) in intestinal luminal samples (collected from duodenum, jejunum and ileum), and the presence of anti-*Eimeria* IgG, IL-10 and IFN-γ in plasma samples, were determined using the ELISA procedures described in detail elsewhere (Ren et al., 2020).

### 2.4 Statistical analysis

Differences in response traits due to SID M+C, coccidiosis, and anti-IL-10 were tested using a 3-way ANOVA (SAS software version 9.2, SAS Institute Inc.). Levene's test was performed to examine the assumption of equal variance among the experimental treatments. Log transformations were performed on luminal anti-*Eimeria* IgA titers, total IgA, and IFN-γ concentrations, and square root transformations were performed on luminal IL-10 concentrations to meet the assumptions of ANOVA. Duncan's multiple range post hoc tests were performed if any of the main effects or interactions were significant. The results were considered significantly different at *P* ≤ 0.05, and a trend at *P* ≤ 0.1. All data were reported as means ± SEM.

## 3 Results

### 3.1 Growth performance

**Fig. 1:**
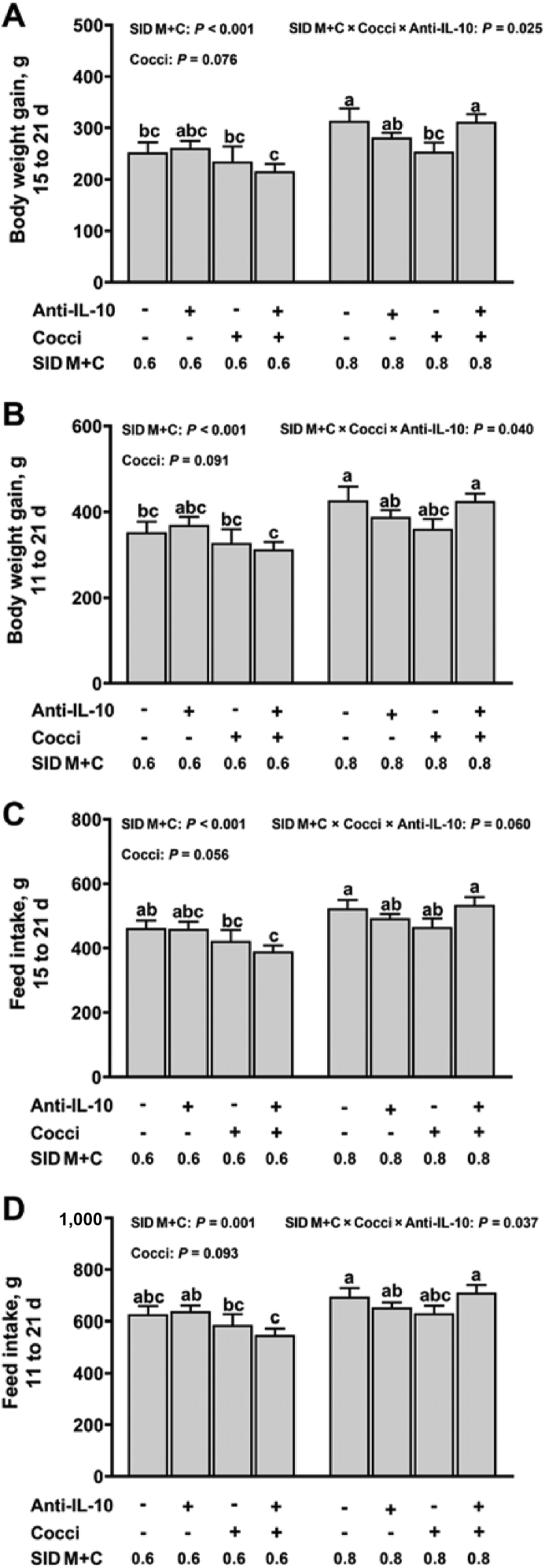
Growth performance of broilers in control conditions or infected with *Eimeria*. Data are means ± SEM. Labeled means without a common letter differ, *P* ≤ 0.05. SID M+C = standardized ileal digestible methionine + cysteine; Cocci = coccidiosis; Anti-IL-10 = egg yolk antibody to interleukin-10 peptide (val-leu-pro-arg-ala-met-gln-thr, vlpramqt).

During the periods of d 15 to 21 and d 11 to 21, interactions (*P* ≤ 0.05) were detected among SID M+C, coccidiosis, and anti-IL-10 effects on body weight gain and feed intake (Fig. 1). Briefly; (1) the highest body weight gain and feed intake were achieved in PBS-gavaged broilers fed 0.8% SID M+C plus no anti-IL-10, and in *Eimeria*-gavaged broilers fed 0.8% SID M+C plus anti-IL-10, (2) in *Eimeria*-gavaged broilers, the supplementation of anti-IL-10 had no effect on body weight gain and feed intake when fed with 0.6% SID M+C, but increased body weight gain (d 15 to 21) when fed with 0.8% SID M+C. No interaction effects (*P* > 0.05) were observed for feed-to-gain ratio during the periods of d 15 to 21 and d 11 to 21 and for any growth performance parameters during the periods of d 11 to 14. However, broilers fed 0.8% SID M+C tended to have an increased body weight gain (main effect, d 11 to 14, *P* = 0.081, Appendix Table 3), and had decreased feed-to-gain ratio (main effect; d 11 to 14, *P* = 0.019; d 11 to 21, *P* = 0.019; Appendix Table 4), when compared to broilers fed 0.6% SID M+C.

### 3.2 Fecal oocyst shedding and intestinal lesion score

**Fig. 2:**
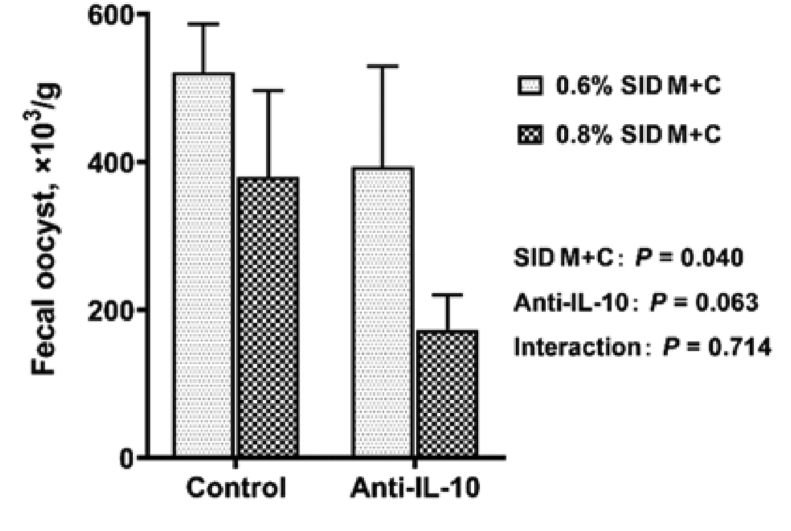
Fecal oocyst counts of broilers in control conditions or infected with *Eimeria*. Data are means ± SEM. SID M+C = standardized ileal digestible methionine + cysteine; Anti-IL-10 = egg yolk antibody to interleukin-10 peptide (val-leu-pro-arg-ala-met-gln-thr, vlpramqt).

**Fig. 3:**
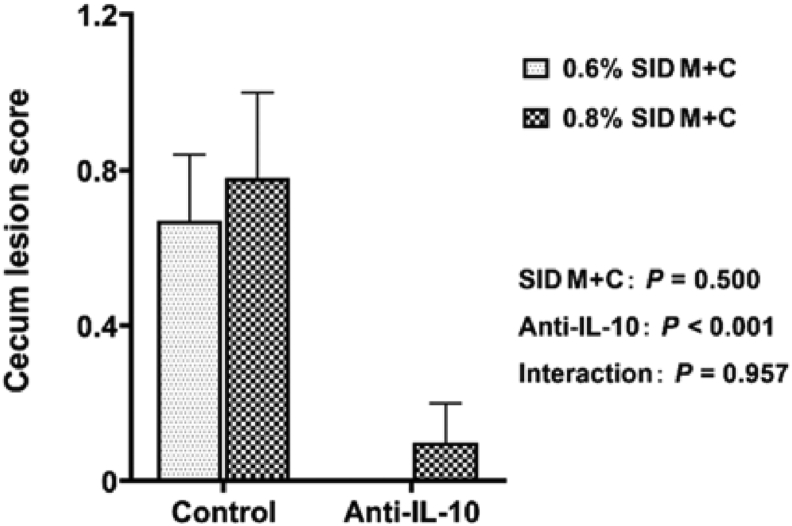
Cecum lesion scores of broilers in control conditions or infected with *Eimeria*. Data are means ± SEM. SID M + C = standardized ileal digestible methionine + cysteine; Anti-IL-10 = egg yolk antibody to interleukin-10 peptide (val-leu-pro-arg-ala-met-gln-thr, vlpramqt).

*Eimeria* oocyst shedding and intestine lesions were detected in *Eimeria*-gavaged but not PBS-gavaged broilers. On d 21, no interaction effects (*P* > 0.05) were observed between SID M+C and anti-IL-10 for fecal oocyst counts (Fig. 2) and intestinal lesion scores (cecum, Fig. 3; duodenum, jejunum, and ileum, Appendix Fig. 1). However, broilers fed 0.8% SID M+C had decreased (main effect, *P* = 0.040) fecal oocyst counts when compared to those fed 0.6% SID M+C. Broilers fed with anti-IL-10 tended to have decreased (main effect, *P* = 0.063) fecal oocyst counts and had decreased cecum lesion scores (main effect, *P* < 0.001) when compared to those fed with no anti-IL-10.

### 3.3 Intestinal luminal T-IgA

**Fig. 4:**
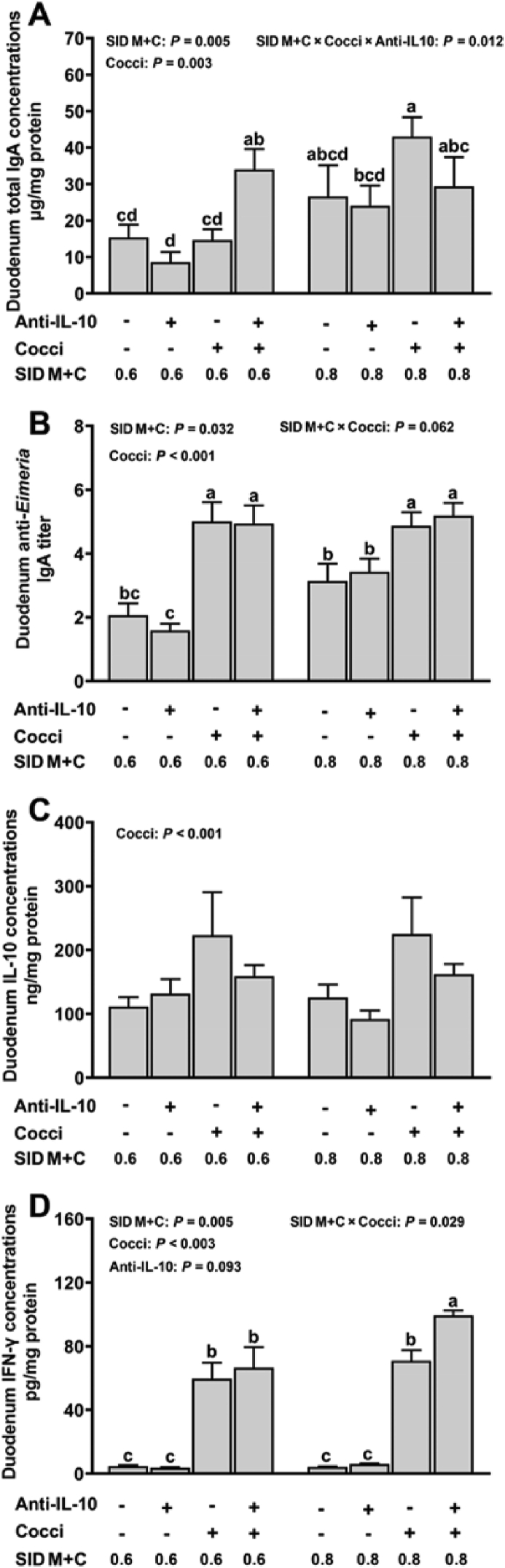
Duodenum T-IgA (A), anti-*Eimeria* IgA (B), IL-10 (C), and IFN-γ (D) concentrations of broilers in control conditions or infected with *Eimeria*. Data are means ± SEM. Labeled means without a common letter differ, *P* ≤ 0.05. SID M+C = standardized ileal digestible methionine + cysteine; Cocci = coccidiosis; Anti-IL-10 = egg yolk antibody to interleukin-10 peptide (val-leu-pro-arg-ala-met-gln-thr, vlpramqt); IFN-γ = interferon gamma.

**Fig. 5:**
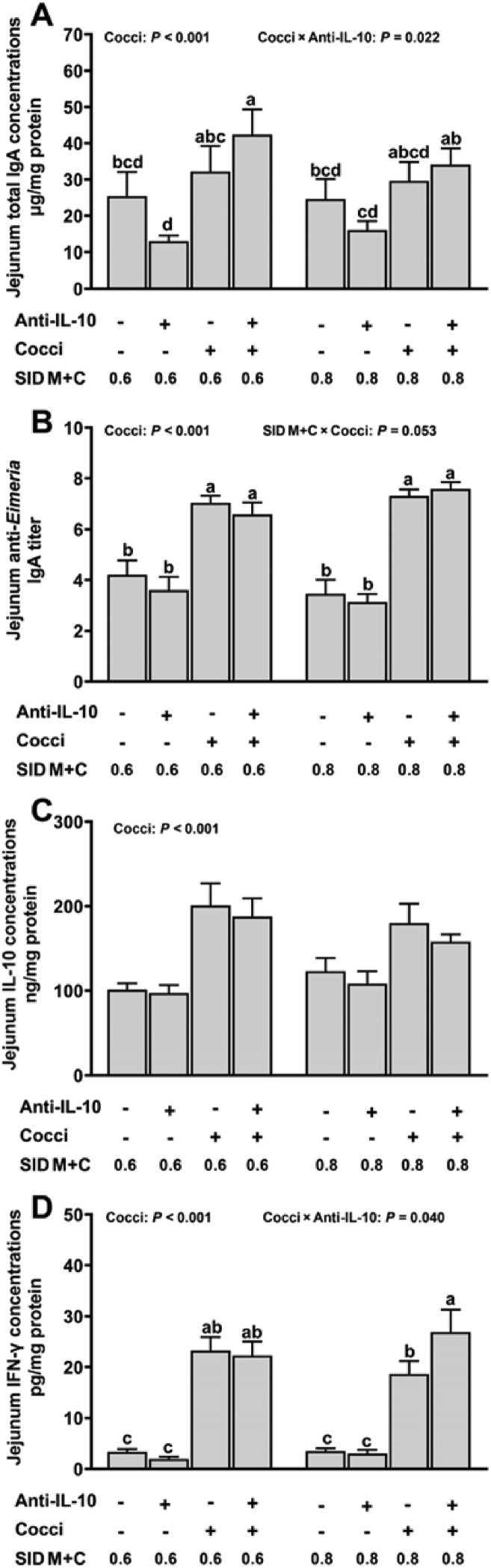
Jejunum T-IgA (A), anti-*Eimeria* IgA (B), IL-10 (C), and IFN-γ (D) concentrations of broilers in control conditions or infected with *Eimeria*. Data are means ± SEM. Labeled means without a common letter differ, *P* ≤ 0.05. Cocci = coccidiosis; Anti-IL-10 = egg yolk antibody to interleukin-10 peptide (val-leu-pro-arg-ala-met-gln-thr, vlpramqt); SID M+C = standardized ileal digestible methionine + cysteine; IFN-γ = interferon gamma.

**Fig. 6:**
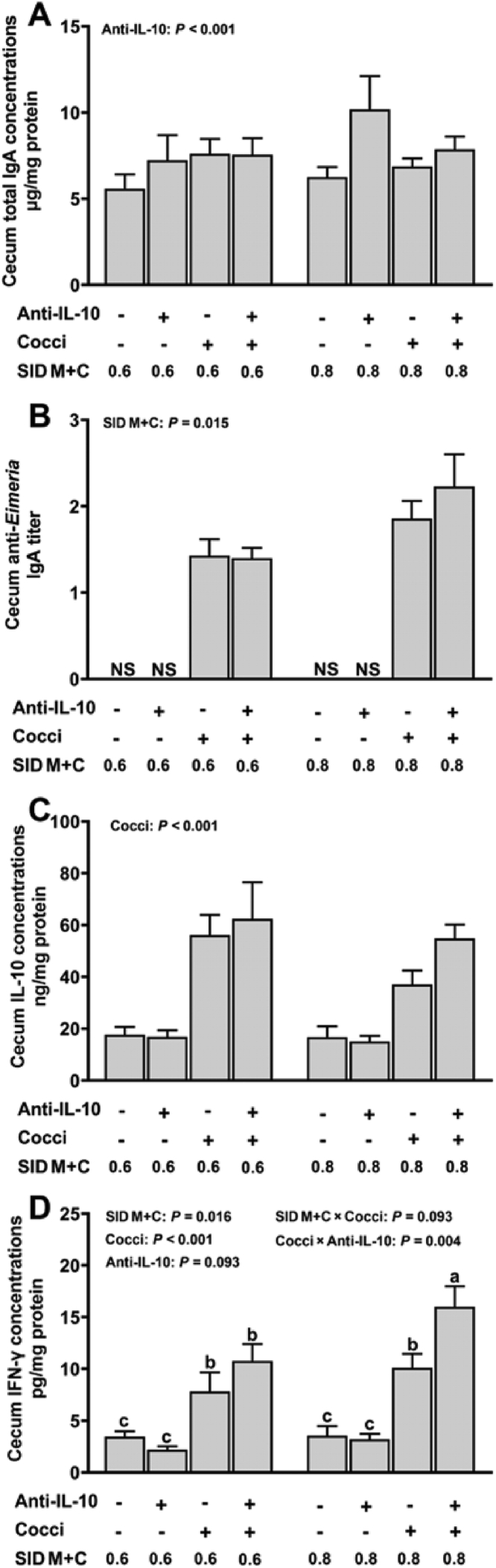
Cecum T-IgA (A), anti-*Eimeria* IgA (B), IL-10 (C), and IFN-γ (D) concentrations of broilers in control conditions or infected with *Eimeria*. Data are means ± SEM. Labeled means without a common letter differ, *P* ≤ 0.05. Anti-IL-10 = egg yolk antibody to interleukin-10 peptide (val-leu-pro-arg-ala-met-gln-thr, vlpramqt); Cocci = coccidiosis; SID M+C = standardized ileal digestible methionine + cysteine; IFN-γ = interferon gamma.

In the duodenal lumen, an interaction among SID M+C, coccidiosis, and anti-IL-10 was observed for T-IgA (*P* = 0.012, Fig. 4A). Briefly, (1) the highest duodenum luminal T-IgA concentration was observed in *Eimeria*-gavaged broilers fed with 0.8% SID M+C plus no anti-IL-10, (2) in *Eimeria*-gavaged broilers, the supplementation of anti-IL-10 increased duodenum luminal T-IgA concentration when fed diets with 0.6% but not 0.8% SID M+C, (3) in *Eimeria*-gavaged broilers fed diets with no anti-IL-10, duodenum luminal T-IgA concentration was increased with a greater concentration of dietary SID M+C. In jejunum luminal, an interaction between coccidiosis and anti-IL-10 was observed for T-IgA (*P* = 0.022, Fig. 5A). Regardless of dietary SID M+C levels, coccidiosis induced increased jejunum luminal T-IgA concentration only with the presence of anti-IL-10. No interaction effect (*P* > 0.05) was observed for cecum luminal T-IgA concentration. However, cecum luminal T-IgA concentration was increased by the supplementation of anti-IL-10 (main effect, *P* < 0.001, Fig. 6A).

### 3.4 Intestine and plasma levels of anti-Eimeria antibody

**Fig. 7:**
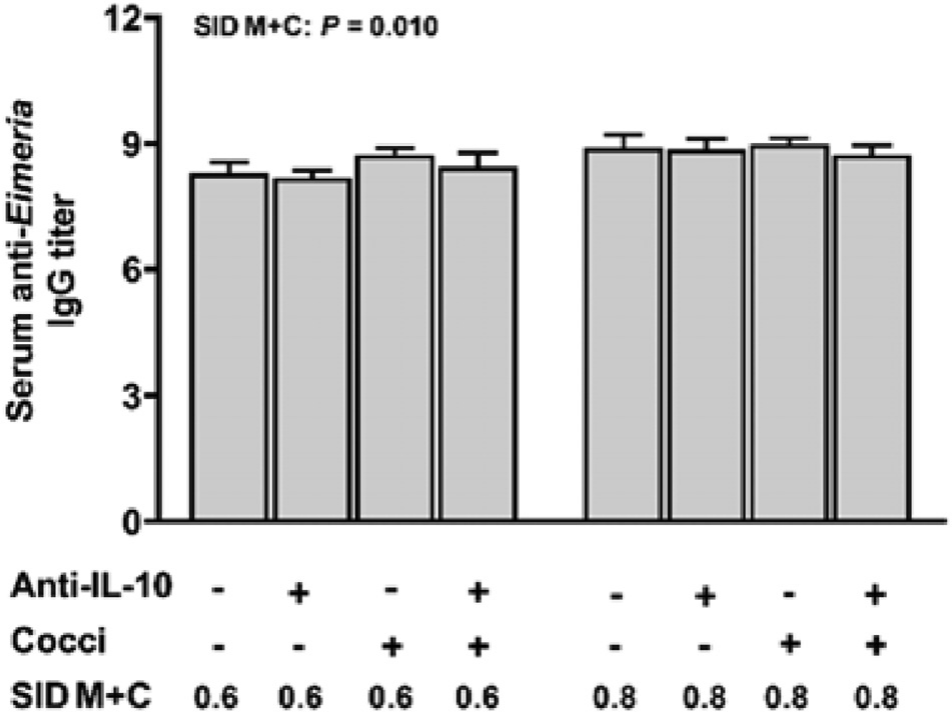
Serum anti-*Eimeria* IgG concentrations of broilers in control conditions or infected with *Eimeria*. Data are means ± SEM. SID M+C = standardized ileal digestible methionine + cysteine; Anti-IL-10 = egg yolk antibody to interleukin-10 peptide (val-leu-pro-arg-ala-met-gln-thr, vlpramqt); Cocci = coccidiosis.

No interaction effects (*P* > 0.05) were observed for intestinal luminal anti*-Eimeria* IgA titer and plasma anti-*Eimeria* IgG titer. Duodenum (Fig. 4B) and jejunum (Fig. 5B) luminal anti-*Eimeria* IgA titer were increased in broilers with coccidiosis (main effect, *P* < 0.001). In the cecum lumen (Fig. 6B), anti-*Eimeria* IgA was detected only in *Eimeria*-gavaged broilers but not PBS-gavaged broilers. Broilers fed diets with 0.8% SID M+C has increased duodenum (main effect, *P* = 0.032) and cecum (main effect, *P* = 0.015) luminal anti*-Eimeria* IgA titers and plasma anti*-Eimeria* IgG titers (main effect, *P* = 0.010, Fig. 7) when compared to those fed diets with 0.6% SID M+C.

### 3.5 Intestine and plasma levels of IL-10 and IFN-γ

No interaction effects (*P* > 0.05) were observed for intestinal luminal (duodenum, Fig. 4C; jejunum, Fig. 5C; cecum, Fig. 6C) and plasma (Appendix Fig. 2) levels of IL-10. However, intestinal luminal IL-10 concentrations were increased (main effect, *P* < 0.001) in broilers with coccidiosis; regardless of dietary SID M+C and anti-IL-10 supplements. Interaction effects were detected on duodenum (SID M+C × coccidiosis, *P* = 0.029, Fig. 4D), jejunum (coccidiosis × anti-IL-10, *P* = 0.040, Fig. 5D), and cecum (coccidiosis × anti-IL-10, *P* = 0.004, Fig. 6D) luminal IFN-γ concentrations. Briefly; (1) intestinal luminal IFN-γ was increased in *Eimeria*-gavaged broilers, regardless of dietary SID M+C and anti-IL-10 treatments, (2) in *Eimeria*-gavaged broilers, anti-IL-10 increased intestinal luminal IFN-γ concentrations in broilers fed diets with 0.8% SID M+C but not 0.6% SID M+C. Plasma IFN-γ (*P* > 0.05, Appendix Fig. 3) concentrations were not affected by the factors studied.

## 4 Discussion

Oral antibodies to IL-10 have previously been shown to confer protection against intestinal lesions and growth suppression in broilers with coccidiosis (Arendt et al., 2016; Lessard et al., 2020; Rasheed et al., 2020; Sand et al., 2016). In the present study, the immunotherapeutic effects of anti-IL-10 to coccidiosis were confirmed using a model of *Eimeria* infection. The benefit of anti-IL-10 during *Eimeria* infection suggests that *Eimeria* may use host IL-10 to exploit or evade the intestinal immune response (Arendt et al., 2019a, Arendt et al., 2019b). Currently, the mode of action by which *Eimeria* hijacks host IL-10 to benefit its pathogenesis has not been established. Further research is warranted to dissect the specific context and mechanisms in which the current anti-IL-10 antibody exerts its actions. Interestingly, anti-IL-10 increased the growth performance of *Eimeria*-infected broilers, only when they were fed with levels of SID M+C (i.e., 0.8%) that are known to be sufficient for growth as described by current nutritional recommendations. For example, the recommendation for d 11 to 21 Ross broilers is 0.80% digestible M+C (Aviagen, UK) but AMINOChick 2.0 (Evonik Operations GmbH, Germany) recommends SID M+C of 0.83% for d 11 to 21 male broilers. Our previous data repeatedly supports that dietary SID M+C deficiency can delay or impair the development of intestinal immunity in birds infected and possibly vaccinated with *Eimeria* (Ren et al., 2020; Tsiagbe et al., 1987a, 1987b). Seemly, dietary supplementation of SID M+C will potentiate the ability of anti-IL-10 to increase resistance to the *Eimeria* infection in an additive or synergistic manner. This may suggest slightly higher SID M+C requirement when *Eimeria* challenged broilers are treated with anti-IL-10 antibodies and should be investigated in future research.

A number of studies have attempted to understand the Immune defense mechanism for life-long protection against *Eimeria* (Fatoba and Adeleke, 2018). The use of athymic animals has shown that B cells are critical to *Eimeria* immunity (resistance) (Rose, 1987; Rose and Hesketh, 1979). When serum of *Eimeria*-infected chicks was transferred to naïve chicks, protection against coccidiosis was observed (Rose and Hesketh, 1979). We also conducted experiments to investigate the efficacy of anti-*Eimeria* antibody for protection against coccidiosis. In an *Eimeria* infection model, chicks fed control egg yolk antibody lost 5% to 8% more body weight gain when compared to those fed anti-*Eimeria* egg yolk antibody. The chicks infected with *Eimeria* and fed the anti-*Eimeria* antibody had growth performance equal to the chicks not infected and fed control or anti-*Eimeria* antibody (unpublished data). These results show that anti-*Eimeria* antibody at the level of the intestinal lumen is an important immune defense against *Eimeria* infection. In the literature, intestinal luminal antibody responses are poorly studied and studies on *Eimeria* infection and antibody responses have largely depended on measures of serum antibody levels. Thus, a series of ELISA assays were developed in our lab to illustrate both serum and intestinal luminal antibody responses in *Eimeria*-infected broilers. As was reported in our previous studies (Ren et al., 2020), increasing dietary SID M+C levels resulted in increased serum anti-*Eimeria* IgG and duodenum and cecum anti-*Eimeria* IgA concentrations. Notably, in the current coccidiosis model, *Eimeria*-infection significantly induced intestinal luminal anti-*Eimeria* IgA production regardless of dietary anti-IL-10 and SID M+C levels. However, *Eimeria*-infection only induced duodenum and jejunum luminal T-IgA production if broilers were fed diets with anti-IL-10. So, it is possible that anti-IL-10 may exert its actions against *Eimeria*-infection by increasing the production of intestinal T-IgA (Virdi et al., 2019). Because the intestinal luminal T-IgA concentrations were not always consistent with the growth performance data, the immunological value of the increased intestinal luminal T-IgA will need to be further investigated.

Several studies have extensively measured mRNA of many cytokine genes in intestinal intraepithelial lymphocytes (Hong et al., 2006a, 2006b) in coccidiosis challenges to determine the natural immune response to these parasites. For example, the expression of mRNA of cytokines that were increased after *Eimeria* challenge include IFN-γ, IL-1β, IL-3, IL-6, IL-8, IL-10, L12, IL-13, IL-15, IL-17, IL-18, granulocyte macrophage colony-stimulating factor, lymphotactin, macrophage migration inhibitory factor, and K203 (Hong et al., 2006b). Among the cytokine mRNA measured, the only ones unchanged were IFN-α, transforming growth factor β4 and K60 (Hong et al., 2006b). The authors deduced that the immune responses to *Eimeria* infections tend to involve both Th1 and Th2 cytokines and flow cytometry supported this as they found an increase in multiple T cell subpopulations. Obviously, simply measuring a breadth of cytokine mRNA responses during *Eimeria* infection provides limited information on immune mechanisms (Mtshali and Adeleke, 2020). Thus, targeting specific cytokine proteins and understanding the interplay between key cytokines is critically important. This strategy was used in a study where the application of IFN-γ and IL-1 proteins were directly used to control *Eimeria* infection (Yun et al., 2000). In another study, IFN-γ, but not IFN-α, protein administration protected against weight loss caused by *Eimeria* and decreased oocyst shedding (Lillehoj and Choi, 1998). Clearly from the experiments described above, IFN-γ is one of the more important cytokines for *Eimeria* resistance (Kim et al., 2019). In the present study, we confirmed that intestinal luminal IFN-γ concentrations were increased in response to *Eimeria* infection. Meanwhile, intestinal luminal presence of IL-10, an anti-inflammatory cytokine which negatively regulates IFN-γ production (Collier et al., 2008), was also increased after the infection. These results are consistent with the idea that maintenance of intestinal luminal IFN-γ levels without the suppressive effects caused by elevated IL-10 levels during *Eimeria* infection will accentuate immune protection without adverse effects on growth performance.

As expected, by binding the luminal IL-10 using oral antibodies, we have allowed increased IFN-γ levels that could alter both the pathogenesis of *Eimeria* and the immune response to it. Importantly, the therapeutic effects of anti-IL-10 which allowed increased luminal IFN-γ concentrations only occurred in broilers fed dietary SID M+C levels of 0.8%. These observations infer that dietary sulfur amino acids are not only essential to growth but for enhanced immune responses, as seen with the increased resistance to *Eimeria* in chicks treated with anti-IL-10. While the exact mechanisms for the enhancement of immune function in broilers fed higher levels of sulfur amino acids is not fully defined, recent literature offers some indication. For example, sulfur amino acids may correct methylation defects, improve antigen presentation, IFN signaling, and macrophage tumor necrosis factor alpha expression (Kharbanda et al., 2013). Due to the seemingly important role sulfur amino acids play in immune function, it could be hypothesized that such nutrients are diverted away from growth towards immune function during health challenges (Kogut and Klasing, 2009). However, these data suggest that dietary limitations in essential nutrients result in deficiencies for both growth and immune responses. This is also supported by previous studies showing that increasing the dietary sulfur amino acids levels above requirements for growth have increased serum IFN-γ levels after infection with infectious bursal disease virus (Maroufyan et al., 2013). In the context of the current results, anti-IL-10 allowed chickens to produce more intestinal IFN-γ and therefore avoid the immune escape of *Eimeria*, but only in the treatments fed the higher level of SID M+C (0.8%). Therefore, dietary supplementation of sulfur amino acids above minimum recommended levels are critical for mounting sufficient immune responses to confer resistance to *Eimeria*, as sulfur amino acids are not always prioritized for immune response (as opposed to growth), when dietary limitations exist (Maroufyan et al., 2013; Rao et al., 2003; Ren et al., 2020; Takahashi et al., 1997; Tsiagbe et al., 1987b).

In the field, current coccidiosis control strategies basically include two approaches: vaccines and antibiotics (Fatoba and Adeleke, 2018; Tensa and Jordan, 2018). However, they each have shortcomings such as the long duration for vaccination efficacy or the emergence of antibiotic resistance (Nataliya et al., 2019; Noack et al., 2019). In the future, if anti-IL-10 is commercialized as a new approach for coccidiosis control (Cook et al., 2016), then considerable benefits will be obtained by providing optimal sulfur amino acids as a dietary supplement to enhance the effectiveness of anti-IL-10 (Abdul-Rasheed et al., 2020; Kadykalo et al., 2018).

## 5 Conclusions

Using a subclinical model of *Eimeria* infection, we demonstrated that the immunotherapeutic efficacy of anti-IL-10 to coccidiosis was highly affected by dietary SID M+C levels. The supplementation of anti-IL-10 increased intestinal luminal IFN-γ levels and subsequently decreased intestinal lesions and restored growth performance only if broilers were fed diets with sufficient levels of SID M+C (i.e., 0.8% in the current study). The production of intestinal luminal T-IgA and anti-*Eimeria* IgA in response to *Eimeria* infection were also affected by dietary factors, but the changes were not consistent with the growth performance data. These results underscore the importance of providing optimal nutrient levels, especially those correlated to intestinal IFN-γ production (including but not limited to sulfur amino acids), to support the application of anti-IL-10 against coccidiosis in the poultry industry.

## Author contributions

**Zhouzheng Ren**, **Rose Whelan**, **Mark E. Cook**, **Xiaojun Yang** and **Thomas D. Crenshaw** designed the research. **Zhouzheng Ren**, **Daniel E. Bütz** and **Maria K. Arendt** conducted the animal trial and collected data. **Zhouzheng Ren**, **Jiakun Yan** and **Xiaojun Yang** performed the data analysis. **Zhouzheng Ren**, **Jiakun Yan**, **Rose Whelan**, **Xujie Liao** and **Xiaojun Yang** wrote the paper. **Zhouzheng Ren**, **Xiaojun Yang** and **Thomas D. Crenshaw** have primary responsibility for the final content of the manuscript. All authors read and approved the final manuscript.

## Declaration of competing interest

We declare that we have no financial and personal relationships with other people or organizations that can inappropriately influence our work, and there is no professional or other personal interest of any nature or kind in any product, service and/or company that could be construed as influencing the content of this paper.
