# Supplementary material for: Dietary supplementation of sulfur amino acids improves intestinal immunity to Eimeria in broilers treated with anti-interleukin-10 antibody
Source: Anim Nutr. 2022 Jun 22;10:382–9. doi: 10.1016/j.aninu.2022.06.008 (PMC9356037; doi:10.1016/j.aninu.2022.06.008)
Supplement: Multimedia component 1 [file mmc1.docx]

**Appendix**

**Table 1.** Ingredient and nutrient composition of basal diets for starter and grower phases

| Item | Starter (d 1 to 10) | Grower (d 11 to 21) |
| --- | --- | --- |
| Ingredients, % |  |  |
| Corn | 57.26 | 60.06 |
| Soybean meal, 48% CP | 35.00 | 31.36 |
| Soybean oil | 3.14 | 4.09 |
| Dicalcium phosphate, 22% | 1.76 | 1.63 |
| Calcium carbonate | 0.74 | 0.71 |
| Sodium chloride | 0.37 | 0.37 |
| Choline chloride, 60% | 0.10 | 0.10 |
| DL-Methionine, 99% | 0.31 | 0.06 |
| L-Lysine sulfate, 54.6% | 0.24 | 0.17 |
| L-Threonine, 98.5% | 0.08 | 0.05 |
| Premix ^1^ | 1.00 | 1.00 |
| Sand | - | 0.40 |
| Total | 100.00 | 100.00 |
| Nutritional composition (calculated, as-fed basis) ^2^ | |  |
| AMEn, kcal/kg | 3,008 | 3,086 |
| Crude protein, % | 21.74 | 20.00 |
| SID Methionine, % | 0.59 | 0.33 |
| SID Cysteine, % | 0.29 | 0.27 |
| SID Methionine + Cysteine, % | 0.88 | 0.60 |
| SID Lysine, % | 1.18 | 1.05 |
| SID Threonine, % | 0.77 | 0.69 |

AMEn = nitrogen-corrected apparent metabolizable energy; SID = standardized ileal digestible.

^1^ Supplied per kilogram of diet: copper, 15 mg; iron, 40 mg; zinc, 100 mg; manganese, 100 mg; selenium, 0.35 mg; iodine, 1 mg; vitamin A, 10,000 IU; vitamin D_3_, 5,000 IU; vitamin E, 80 IU; vitamin K, 3 mg; vitamin B_1_, 3 mg; vitamin B_2_, 9 mg; vitamin B_6_, 4 mg; vitamin B_12_, 0.02 mg; nicotinic acid, 60 mg; pantothenic acid, 15 mg; biotin, 0.15 mg; folic acid, 2 mg.

^2^ SID levels were calculated based on poultry specific digestibility coefficients provided by Evonik Operations GmbH (Hanau, Germany).

**Table 2.** Analyzed nutrient and anti-IL-10 levels of experimental diets (%, unless noted, as-fed basis)

| Item | Calculated | Analyzed ^1^ | | | | |
| --- | --- | --- | --- | --- | --- | --- |
|  |  | 0.6% SID M+C | |  | 0.8% SID M+C | |
|  |  | Without anti-IL-10 | With anti-IL-10 |  | Without anti-IL-10 | With anti-IL-10 |
| Crude protein | 20.00 | 19.32 | 18.80 |  | 19.42 | 19.49 |
| Methionine | 0.35/0.55 | 0.31 | 0.32 |  | 0.48 | 0.48 |
| Cysteine | 0.33 | 0.30 | 0.30 |  | 0.30 | 0.30 |
| Methionine + Cysteine | 0.68/0.88 | 0.61 | 0.62 |  | 0.78 | 0.78 |
| Lysine | 1.15 | 1.17 | 1.14 |  | 1.17 | 1.16 |
| Threonine | 0.80 | 0.74 | 0.74 |  | 0.74 | 0.75 |
| Arginine | 1.32 | 1.28 | 1.26 |  | 1.29 | 1.28 |
| Isoleucine | 0.84 | 0.81 | 0.81 |  | 0.82 | 0.82 |
| Leucine | 1.72 | 1.63 | 1.62 |  | 1.64 | 1.64 |
| Valine | 0.93 | 0.90 | 0.90 |  | 0.92 | 0.91 |
| Histidine | 0.53 | 0.51 | 0.51 |  | 0.51 | 0.51 |
| Phenylalanine | 0.99 | 0.94 | 0.94 |  | 0.95 | 0.95 |
| Glycine | 0.82 | 0.77 | 0.77 |  | 0.78 | 0.77 |
| Serine | 0.98 | 0.91 | 0.91 |  | 0.91 | 0.91 |
| Proline | 1.17 | 1.14 | 1.12 |  | 1.13 | 1.12 |
| Alanine | 1.00 | 0.93 | 0.94 |  | 0.94 | 0.94 |
| Aspartate aminotransferase | 2.04 | 1.90 | 1.89 |  | 1.92 | 1.91 |
| Glutamic acid | 3.58 | 3.39 | 3.38 |  | 3.39 | 3.39 |
| Anti-IL-10, μg/kg | 300 | - | 377 |  | - | 371 |

SID M+C = standardized ileal digestible methionine + cysteine; Anti-IL-10 = egg yolk antibody to interleukin-10 peptide.

^1^ Amino acid content of treatment diets were analyzed by ion-exchange chromatography (AA analyzer LC 3000, Biotronic, Maintal, Germany). The level of anti-IL-10 was analyzed by a lab-developed enzyme-linked immunosorbent assay as described in the text.

**Table 3.** Body weight gain and feed intake of broilers (d 11 to 14)

| SID M+C | Cocci | Anti-IL-10 | Body weight gain, g | Feed intake, g |
| --- | --- | --- | --- | --- |
| 0.6% | - | - | 99 | 165 |
| 0.6% | - | + | 108 | 180 |
| 0.6% | + | - | 93 | 166 |
| 0.6% | + | + | 97 | 161 |
| 0.8% | - | - | 113 | 171 |
| 0.8% | - | + | 107 | 164 |
| 0.8% | + | - | 106 | 167 |
| 0.8% | + | + | 114 | 179 |
| SEM |  |  | 3 | 3 |
|  |  |  |  |  |
| *P*-value |  |  |  |  |
| SID M+C |  |  | 0.081 | 0.714 |
| Challenge |  |  | 0.466 | 0.813 |
| Anti-IL-10 |  |  | 0.534 | 0.617 |
| SID M+C × Challenge |  |  | 0.457 | 0.270 |
| SID M+C × Anti-IL-10 |  |  | 0.630 | 0.854 |
| Challenge × Anti-IL-10 |  |  | 0.729 | 0.989 |
| SID M+C × Challenge × Anti-IL-10 |  |  | 0.439 | 0.156 |

SID M+C = standardized ileal digestible methionine + cysteine; Cocci = coccidiosis; Anti-IL-10 = egg yolk antibody to interleukin-10 peptide (val-leu-pro-arg-ala-met-gln-thr, vlpramqt).

**Table 4.** Feed-to-gain ratio of broilers in control conditions or infected with *Eimeria*

| SID M+C | Cocci | Anti-IL-10 | d 11 to 14, g:g | d 15 to 21, g:g | d 11 to 21, g:g |
| --- | --- | --- | --- | --- | --- |
| 0.6% | - | - | 1.74 | 1.86 | 1.81 |
| 0.6% | - | + | 1.69 | 1.78 | 1.75 |
| 0.6% | + | - | 1.81 | 1.93 | 1.85 |
| 0.6% | + | + | 1.69 | 1.83 | 1.77 |
| 0.8% | - | - | 1.56 | 1.71 | 1.66 |
| 0.8% | - | + | 1.58 | 1.76 | 1.69 |
| 0.8% | + | - | 1.68 | 1.87 | 1.78 |
| 0.8% | + | + | 1.59 | 1.72 | 1.68 |
| SEM |  |  | 0.03 | 0.03 | 0.02 |
|  |  |  |  |  |  |
| *P*-value |  |  |  |  |  |
| SID M+C |  |  | 0.019 | 0.145 | 0.019 |
| Challenge |  |  | 0.404 | 0.286 | 0.297 |
| Anti-IL-10 |  |  | 0.258 | 0.236 | 0.183 |
| SID M+C × Challenge |  |  | 0.812 | 0.932 | 0.750 |
| SID M+C × Anti-IL-10 |  |  | 0.735 | 0.750 | 0.586 |
| Challenge × Anti-IL-10 |  |  | 0.438 | 0.370 | 0.321 |
| SID M+C × Challenge × Anti-IL-10 |  |  | 0.815 | 0.427 | 0.471 |

SID M+C = standardized ileal digestible methionine + cysteine; Cocci = coccidiosis; Anti-IL-10 = egg yolk antibody to interleukin-10 peptide (val-leu-pro-arg-ala-met-gln-thr, vlpramqt).


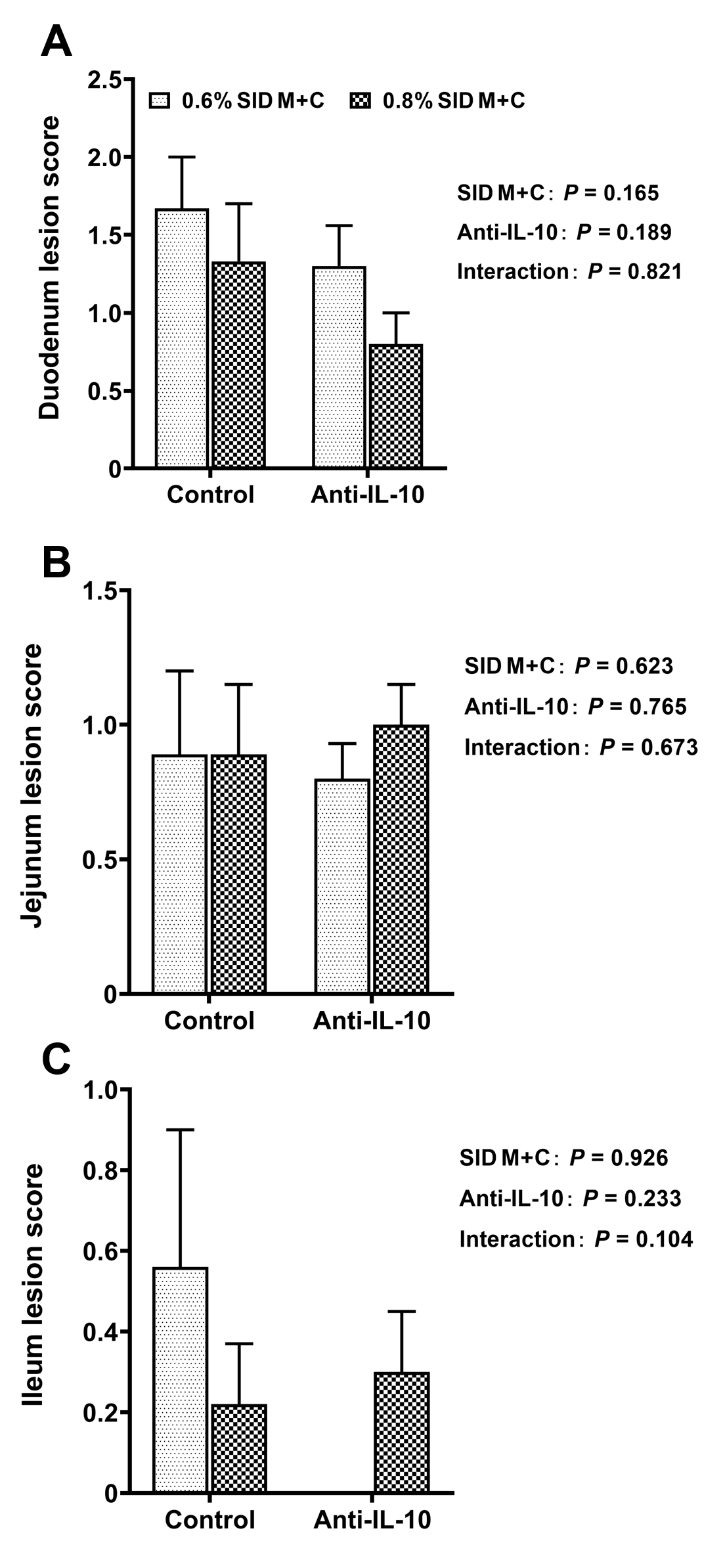


**Fig. 1. Duodenum (A), Jejunum (B), and ileum (C) lesion scores of broilers in control conditions or infected with *Eimeria*.** Data are means ± SEM. SID M+C = standardized ileal digestible methionine + cysteine; Anti-IL-10 = egg yolk antibody to interleukin-10 peptide (val-leu-pro-arg-ala-met-gln-thr, vlpramqt).


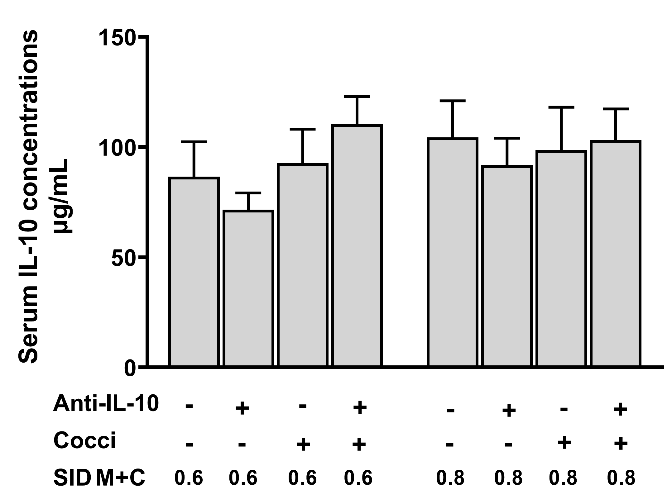


**Fig. 2. Serum IL-10 concentrations of broilers in control conditions or infected with *Eimeria*.** Data are means ± SEM. Anti-IL-10 = egg yolk antibody to interleukin-10 peptide (val-leu-pro-arg-ala-met-gln-thr, vlpramqt); Cocci = coccidiosis; SID M+C = standardized ileal digestible methionine + cysteine.


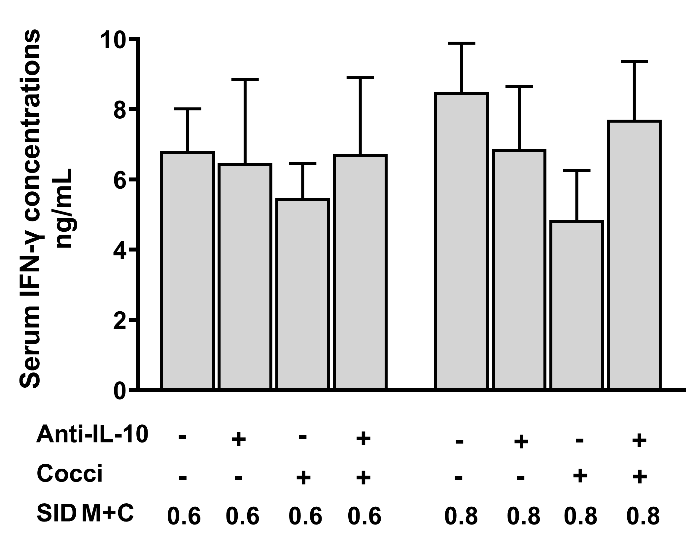


**Fig. 3. Serum IFN-γ concentrations of broilers in control conditions or infected with *Eimeria*.** Data are means ± SEM. IFN-γ = interferon gamma; Anti-IL-10 = egg yolk antibody to interleukin-10 peptide (val-leu-pro-arg-ala-met-gln-thr, vlpramqt); Cocci = coccidiosis; SID M+C = standardized ileal digestible methionine + cysteine.
